# Palmitoylethanolamide in Postmenopausal Metabolic Syndrome: Current Evidence and Clinical Perspectives

**DOI:** 10.3390/nu16244313

**Published:** 2024-12-13

**Authors:** Alessandro Medoro, Sergio Davinelli, Federica Fogacci, Stefania Alfieri, Domenico Tiso, Arrigo F. G. Cicero, Giovanni Scapagnini

**Affiliations:** 1 Department of Medicine and Health Sciences “V.Tiberio”, University of Molise, 86100 Campobasso, Italy; alessandro.medoro@unimol.it (A.M.); giovanni.scapagnini@unimol.it (G.S.); 2 Hypertension and Cardiovascular Risk Research Unit, Medical and Surgical Sciences Department, Alma Mater Studiorum University of Bologna, 40138 Bologna, Italy; federica.fogacci@studio.unibo.it (F.F.); arrigo.cicero@unibo.it (A.F.G.C.); 3 Italian Nutraceutical Society (SINut), 40138 Bologna, Italy; 4 Centro Mediprò, 40068 San Lazzaro di Savena, Italy; s.alfieri@medipro.it; 5 Clinical Nutrition, “Villa Maria” Hospital, 47921 Rimini, Italy; dottortiso@gmail.com; 6 Cardiovascular Medicine Unit, IRCCS Azienda Ospedaliero-Universitaria di Bologna, 40138 Bologna, Italy

**Keywords:** palmitoylethanolamide, menopause, metabolic syndrome, PPAR-α, insulin resistance, weight management, appetite regulation, lifestyle interventions

## Abstract

Menopause leads to a decline in estrogen levels, resulting in significant metabolic alterations that increase the risk of developing metabolic syndrome—a cluster of conditions including central obesity, insulin resistance, dyslipidemia, and hypertension. Traditional interventions such as hormone replacement therapy carry potential adverse effects, and lifestyle modifications alone may not suffice for all women. This review explores the potential role of palmitoylethanolamide (PEA), an endogenous fatty acid amide, in managing metabolic syndrome during the postmenopausal period. PEA primarily acts by activating peroxisome proliferator-activated receptor-alpha (PPAR-α), influencing lipid metabolism, energy homeostasis, and inflammation. Evidence indicates that PEA may promote the browning of white adipocytes, enhancing energy expenditure and reducing adiposity. It also improves lipid profiles by boosting fatty acid oxidation and decreasing lipid synthesis, potentially lowering low-density lipoprotein cholesterol and triglyceride levels while increasing high-density lipoprotein cholesterol. Additionally, the anti-inflammatory properties of PEA enhance insulin sensitivity by reducing pro-inflammatory cytokines that interfere with insulin signaling. PEA may aid in weight management by influencing appetite regulation and improving leptin sensitivity. Furthermore, its neuroprotective effects may address the mood disturbances and cognitive decline associated with menopause. Given these multifaceted biological activities and a favorable safety profile, PEA may represent a promising non-pharmacological supplement for managing metabolic syndrome in postmenopausal women. However, further large-scale clinical studies are necessary to establish its efficacy, optimal dosing, and long-term safety. If validated, PEA could become an integral part of strategies to improve metabolic and neuropsychological health outcomes in this population.

## 1. Introduction

Menopause marks a significant physiological transition in a woman’s life, typically occurring between the ages of 45 and 55, characterized by the permanent cessation of menstruation due to the loss of ovarian follicular activity [1]. This transition encompasses a complex interplay of hormonal changes that have widespread effects on various body systems. The decline in estrogen and progesterone levels during menopause leads to a cascade of metabolic alterations, increasing susceptibility to several chronic conditions, most notably metabolic syndrome [2].

Metabolic syndrome is a group of linked metabolic risk factors, including central obesity, insulin resistance, dyslipidemia, and hypertension. One of the primary indicators is an increased waist circumference—exceeding 40 inches in men and 35 inches in women—which reflects central obesity. Another criterion is elevated serum triglyceride levels of 150 mg/dL or higher. Additionally, the syndrome involves reduced levels of high-density lipoprotein (HDL) cholesterol, specifically less than 40 mg/dL in men or less than 50 mg/dL in women. Elevated fasting glucose levels of 100 mg/dL or greater also represent a significant marker. Lastly, higher blood pressure readings, with a systolic pressure of 130 mm Hg or above or a diastolic pressure of 85 mm Hg or above, contribute to the diagnosis of metabolic syndrome [3]. These factors synergistically increase the risk of developing cardiovascular diseases (CVDs) and type 2 diabetes mellitus (T2DM), which are leading causes of morbidity and mortality worldwide. The prevalence of metabolic syndrome increases during the postmenopausal period, suggesting a pivotal role of hormonal changes in its pathogenesis [4]. The decline in estrogen levels during menopause disrupts the protective metabolic functions previously mediated by estrogens. Estrogens influence lipid metabolism by improving lipid profiles—reducing low-density lipoprotein (LDL) cholesterol and increasing high-density lipoprotein (HDL) cholesterol—and enhance insulin sensitivity by modulating glucose homeostasis [5].

Additionally, estrogen promotes a gynoid pattern of fat distribution, characterized by fat accumulation in the hips and thighs, which is associated with lower metabolic risk compared to abdominal (android) fat distribution. The loss of estrogen leads to a shift toward central adiposity, insulin resistance, dyslipidemia, and increased blood pressure, all of which contribute to the development of metabolic syndrome [6,7]. Furthermore, menopause is associated with a pro-inflammatory state, marked by elevated levels of inflammatory cytokines such as interleukin-6 (IL-6) and tumor necrosis factor-alpha (TNF-α) [8,9]. Chronic low-grade inflammation and oxidative stress are key contributors to insulin resistance, endothelial dysfunction, and vascular damage, contributing to metabolic syndrome onset [7,10].

Considering these complex issues, there is an urgent need for effective solutions to reduce the metabolic alterations that come with menopause. While hormone replacement therapy (HRT) has been employed to alleviate menopausal symptoms and confer cardiometabolic benefits, its use is limited by potential adverse effects, including an increased risk of breast cancer and thromboembolic events [11]. Lifestyle modifications, such as dietary changes and increased physical activity, are fundamental but may not be sufficient for all women. In this context, there is growing interest in exploring non-drug treatments that can help regulate postmenopausal metabolism. Palmitoylethanolamide (PEA) emerges as a promising candidate due to its endogenous nature and multifaceted biological activities. PEA is an endogenous fatty acid amide belonging to the *N*-acylethanolamine family and is involved in various physiological processes, including inflammation, pain perception, and metabolic regulation. PEA primarily exerts its effects by activating peroxisome proliferator-activated receptor-alpha (PPAR-α). This nuclear receptor plays a crucial role in lipid metabolism, energy homeostasis, and inflammation. By activating PPAR-α, PEA may enhance fatty acid oxidation, reduce lipid synthesis, and modulate inflammatory responses [12]. These mechanisms align closely with the metabolic alterations observed during menopause, suggesting that PEA could potentially alleviate the development of metabolic syndrome in postmenopausal women. Its favorable safety profile and minimal side effects make it an attractive option for long-term use, also considering the neuroprotective and analgesic properties, which could address other menopausal symptoms, such as sleep and mood disturbances and neuropathic pain [13]. This review aims to describe the potential role of PEA in managing postmenopausal metabolic syndrome, highlighting evidence from preclinical and clinical studies.

## 2. Menopause and the Consequences of the Estrogen Decline: A Focus on the Metabolic Syndrome

Menopause is a natural biological process signifying the end of a woman’s reproductive capacity, diagnosed retrospectively after 12 consecutive months of amenorrhea without any pathological cause, affecting the total hormonal balance [14]. These hormonal changes during perimenopause are the major cause of metabolic alterations that characterize metabolic syndrome and affect psychological well-being, leading to mood swings, depression, and anxiety. These factors may contribute to unhealthy behaviors such as poor diet, physical inactivity, and smoking, which exacerbate metabolic syndrome [15,16]. Indeed, multiple studies have documented the increased prevalence of metabolic syndrome in postmenopausal women [17,18,19,20,21]. The Third National Health and Nutrition Examination Survey (NHANES III) reported that the prevalence of metabolic syndrome was higher in postmenopausal compared to premenopausal women [22]. Kim et al. found similar results in the Korean population, highlighting the global relevance of this issue [23].

During the premenopausal years, the pulsatile release of gonadotropin-releasing hormone (GnRH) stimulates the anterior pituitary gland to synthesize and release follicle-stimulating hormone (FSH) and luteinizing hormone (LH). FSH, in particular, encourages ovarian follicles to produce estradiol and inhibin B. These hormones provide feedback to the hypothalamus and pituitary gland, modulating the production of GnRH, LH, and FSH. The synchronized and timely release of FSH and LH from the pituitary gland leads to the development of ovarian follicles, ovulation, and menstruation. Inhibin B, produced by ovarian granulosa cells, specifically inhibits the synthesis and secretion of FSH. The menopausal transition, or perimenopause, is characterized by fluctuating hormonal levels, particularly estrogen and progesterone, due to declining ovarian follicular activity. These hormonal fluctuations can span several years before menstruation ceases, leading to physiological and symptomatic changes. After menopause, the depletion of ovarian follicles leads to a decline in the production of estradiol and inhibin B, and ovulation and menstruation cease. The ovaries become less responsive to FSH and LH, and the reduced negative feedback from estradiol and inhibin B on the hypothalamic–pituitary axis results in increased production and release of GnRH, FSH, and LH. Elevated FSH levels are particularly characteristic of the postmenopausal period [4].

The decline in estrogen levels during menopause affects multiple organ systems due to the widespread distribution of estrogen receptors (ERs) throughout the body. Estrogen exerts its effects through two main receptors, ERα and ERβ, expressed in tissues such as the cardiovascular, skeletal, central nervous, and adipose tissue [24]. Reducing estrogen leads to vasomotor symptoms (hot flashes, night sweats), urogenital atrophy, osteoporosis, mood and cognition changes, and alterations in lipid and glucose metabolism [25].

Estrogen influences lipid metabolism by modulating the expression of enzymes and receptors involved in lipid synthesis and clearance. It enhances the expression of LDL receptors in the liver, promoting the clearance of LDL cholesterol from the bloodstream. Estrogen also increases the synthesis of HDL cholesterol, which protects against atherosclerosis. The decline in estrogen during menopause leads to unfavorable lipid profiles, characterized by elevated LDL cholesterol and triglycerides and decreased HDL cholesterol [26]. One of the most significant physiological changes is the shift in body fat distribution. Premenopausal women typically exhibit a gynoid fat distribution, with fat predominantly stored in subcutaneous depots around the hips and thighs. This pattern is associated with a lower risk of metabolic diseases. Postmenopausal women, however, tend to accumulate fat centrally, leading to increased visceral adiposity. Visceral fat is metabolically active and contributes to insulin resistance, dyslipidemia, and a pro-inflammatory state [27].

In glucose metabolism, estrogens enhance insulin sensitivity by improving insulin receptor signaling and glucose uptake in peripheral tissues. They upregulate the expression of glucose transporters and modulate the activity of enzymes involved in glucose metabolism. Estrogen deficiency contributes to insulin resistance, a core component of metabolic syndrome and a precursor to T2DM [28,29]. Menopause is also associated with increased pro-inflammatory cytokines and inflammatory markers, including IL-6, TNF-α, and C-reactive protein (CRP). Adipose tissue, particularly visceral fat, secretes adipokines and cytokines that exacerbate inflammation. Chronic low-grade inflammation promotes insulin resistance through insulin-interfering signaling pathways [30,31]. Estrogen has anti-inflammatory properties, and its decline removes this protective effect, contributing to an imbalanced inflammatory state. Oxidative stress increases during menopause due to decreased antioxidant defenses and increased production of reactive oxygen species (ROS). Oxidative stress damages endothelial cells, leading to endothelial dysfunction—a critical factor in the development of atherosclerosis and hypertension. Estrogen normally enhances antioxidant enzyme expression and activity (principally superoxide dismutase), reducing oxidative damage [32]. The combined effects of dyslipidemia, insulin resistance, inflammation, and oxidative stress significantly elevate the risk of cardiovascular diseases in postmenopausal women [33,34]. Epidemiological studies have shown that the incidence of cardiovascular disease increases markedly after menopause [35,36,37]. The protective effects of estrogen on the vascular endothelium, lipid metabolism, and hemostatic factors are diminished in postmenopausal women, accelerating the atherogenic process [38].

Addressing metabolic syndrome in postmenopausal women requires a multifaceted approach. Lifestyle modifications are the cornerstone of management, emphasizing a balanced diet, regular physical activity, weight management, and smoking cessation [39]. Dietary interventions usually focus on reducing saturated fats, trans fats, and simple sugars while increasing fiber intake. Pharmacological interventions may be necessary for managing specific components of metabolic syndrome, such as antihypertensive agents, lipid-lowering, and antidiabetic drugs. However, the use of HRT remains controversial due to associated risks. Complementary dietary supplements may represent a non-pharmacological approach with favorable safety profiles to provide additional benefits without significant adverse effects [21,40,41].

## 3. Palmitoylethanolamide (PEA): Metabolism and Molecular Targets

PEA is an endogenous lipid mediator first identified in the 1950s. It belongs to the *N*-acylethanolamine (NAE) family, which includes other bioactive lipids such as the endocannabinoid anandamide and its monounsaturated analog oleoylethanolamide. PEA is synthesized from membrane phospholipid precursors in response to physiological and pathological stimuli. It is synthesized through the *N*-acylation-phosphodiesterase pathway, where *N*-acyltransferase (NAT) catalyzes the transfer of a fatty acid to phosphatidylethanolamine, forming *N*-acyl-phosphatidylethanolamine (NAPE). NAPE is then hydrolyzed by NAPE-specific phospholipase D (NAPE-PLD) to produce PEA. The degradation of PEA occurs primarily through fatty acid amide hydrolase (FAAH) and *N*-acylethanolamine acid amidase (NAAA), which hydrolyze PEA back into palmitic acid and ethanolamine [12].

PEA modulates several physiological processes, including cellular metabolism, inflammation, immune responses, neuroprotection, and pain perception. PEA exerts its effects through multiple mechanisms. The primary mechanism involves the activation of PPAR-α, a nuclear receptor that regulates gene expression in lipid metabolism, inflammation, and energy homeostasis and is considered a target for metabolic syndrome. After ligand binding, PPAR-α heterodimerizes with its obligate partner, the retinoic acid X receptor (RXR). This complex then binds to peroxisome proliferator response elements (PPREs), specific DNA sequences located in the promoter regions of target genes [42]. PPAR-α activation induces lipid uptake and catabolism (fatty acid oxidation, in particular, β-oxidation) and the production of apolipoproteins A-I and A-II, thereby diminishing circulating triglyceride and LDL cholesterol and increasing the HDL cholesterol levels. These effects on lipid metabolism are particularly relevant in conditions characterized by dyslipidemia, such as metabolic syndrome. In addition to their anti-dyslipidemic activities, recent in vitro and preclinical data indicate that PPAR-α agonists also have direct vasoprotective effects [43].

The anti-inflammatory effects of PEA are well-documented in various models of inflammation and pain. By activating PPAR-α, PEA inhibits the expression of pro-inflammatory genes, including cyclooxygenase-2 (COX-2), inducible nitric oxide synthase (iNOS), and several cytokines, and promotes the production of anti-inflammatory cytokines. PEA also regulates mast cell degranulation, reducing the release of pro-inflammatory mediators such as histamines, cytokines, and proteases [12,44]. In neuropathic pain models, PEA reduces hyperalgesia and allodynia by modulating glial cell activation and decreasing neuroinflammation involving the cannabinoid receptors. While PEA does not directly bind to cannabinoid receptors CB1 and CB2, it can enhance the levels of the endocannabinoid anandamide by inhibiting FAAH, indirectly influencing the endocannabinoid system (entourage effect). Additionally, PEA may interact with other receptors, such as GPR55 and transient receptor potential vanilloid type 1 (TRPV1) channels, further influencing pain perception and inflammation [12].

## 4. Potential Benefits of PEA in Postmenopausal Metabolic Syndrome

**Figure 1:**
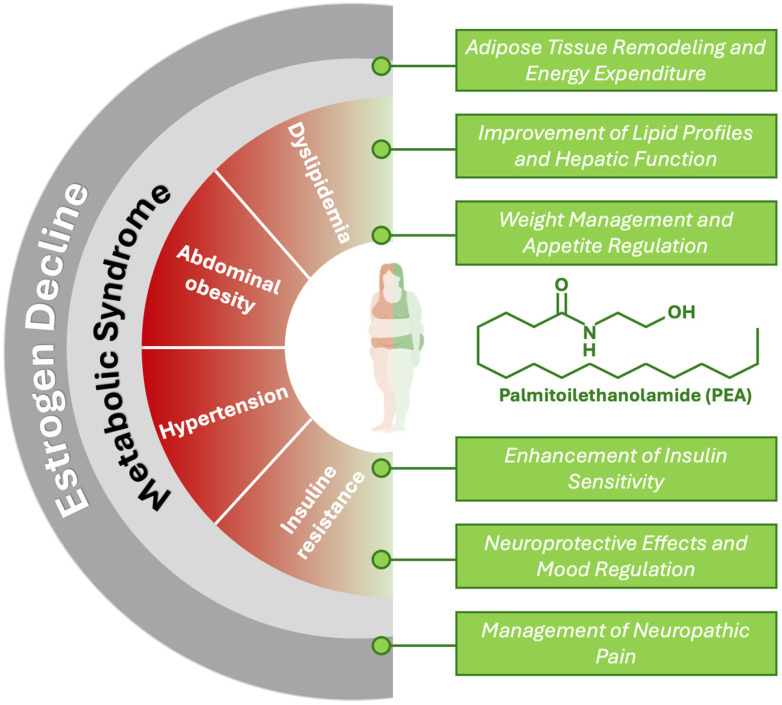
Potential beneficial effects of PEA on clinical features of postmenopausal metabolic syndrome.

The multifaceted mechanisms of PEA in regulating lipid metabolism, inflammation, and energy homeostasis position it as a promising agent for addressing metabolic syndrome in postmenopausal women. The decline in estrogen during menopause leads to metabolic disturbances that PEA may counteract through several pathways (Figure 1).

### 4.1. Adipose Tissue Remodeling and Energy Expenditure

**Figure 2:**
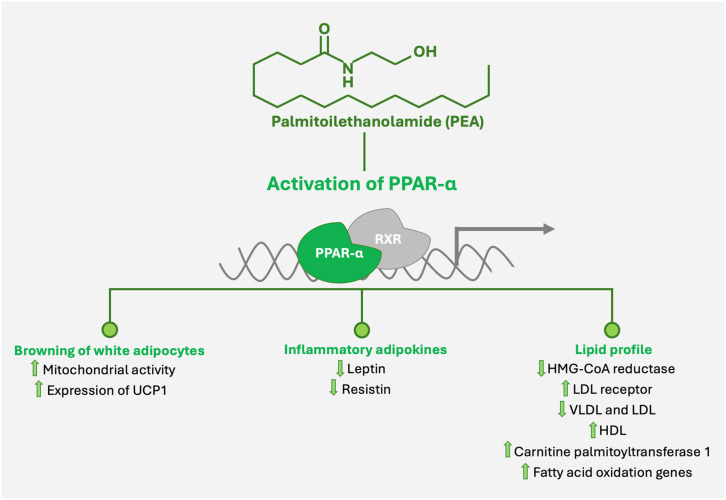
Mechanisms of PEA-induced activation of PPAR-α and its effects on adipocyte browning, inflammatory adipokines, and lipid metabolism. Upward arrows indicate an increase, while downward arrows indicate a decrease.

One significant area of interest is the role of PEA in adipose tissue remodeling. Annunziata et al. demonstrated that PEA may promote the browning of white adipocytes, inducing a white-to-beige conversion through PPAR-α activation [45]. Beige adipocytes possess thermogenic capabilities similar to brown adipose tissue, characterized by increased mitochondrial activity and the expression of uncoupling protein 1 (UCP1), a major contributor to thermogenesis [46] (Figure 2). This process enhances energy expenditure and reduces adiposity, which can mitigate obesity—a key component of metabolic syndrome. For postmenopausal women, who often experience increased central fat accumulation and metabolic alterations, PEA could help counterbalance the reduced metabolic rate associated with aging and hormonal changes [47]. Moreover, PEA may influence adipose tissue modulating adipokine secretion. Inflammatory adipokines such as leptin and resistin are elevated in obesity, contributing to insulin resistance [44]. PEA has been shown to normalize adipokine secretion profiles, decreasing pro-inflammatory adipokines. This shift may enhance insulin sensitivity and reduce systemic inflammation [48,49].

### 4.2. Improvement of Lipid Profiles and Hepatic Function

For postmenopausal women, the risk of developing metabolic syndrome and dyslipidemia increases due to estrogen deficiency, which adversely affects lipid metabolism [16]. By improving hepatic lipid metabolism, PEA could help mitigate these risks. The activation of PPAR-α by PEA may enhance lipid metabolism, potentially leading to improved lipid profiles. The potential influence of PEA on cholesterol homeostasis, such as modulating enzymes involved in cholesterol synthesis and clearance, may contribute to lowering LDL cholesterol levels and improving overall lipid profiles [48]. Studies have indicated that PPAR-α can downregulate 3-hydroxy-3-methylglutaryl-CoA reductase (HMG-CoA reductase)—the rate-limiting enzyme in cholesterol synthesis—and upregulate LDL receptor expression, promoting cholesterol uptake and clearance from the bloodstream [50,51]. Indeed, the activation of PPAR-α influences lipoprotein metabolism by causing a decrease in both very-low-density lipoproteins (VLDLs) synthesized in the liver and LDL while leading to an increase in HDL [52] (Figure 2).

Moreover, PPAR-α activation promotes gene expression in mitochondrial fatty acid oxidation, such as medium-chain acyl-CoA dehydrogenase (MCAD) or long-chain acyl-CoA dehydrogenase (LCAD), thereby reducing lipid accumulation in the liver and adipose tissue [53,54] (Figure 2). Metabolic dysfunction-associated steatotic liver disease (MASLD) is often considered the hepatic manifestation of metabolic syndrome and is strongly associated with insulin resistance and obesity. By enhancing mitochondrial function and promoting efficient fatty acid oxidation, PEA may reduce hepatic steatosis—hepatocyte fat accumulation [55]. Furthermore, PEA enhanced the phosphorylation of AMP-activated protein kinase-alpha and increased the transcription of carnitine palmitoyltransferase 1 in adipose tissue, suggesting an upregulation of ATP-producing catabolic pathways [48].

### 4.3. Enhancement of Insulin Sensitivity

Insulin resistance is a key feature of metabolic syndrome, leading to impaired glucose uptake and hyperglycemia [29]. Chronic low-grade inflammation plays a significant role in developing insulin resistance [56]. The anti-inflammatory effects of PEA may improve insulin sensitivity by reducing the levels of pro-inflammatory cytokines, such as tumor necrosis factor-alpha (TNF-α) and interleukin-6 (IL-6), which interfere with insulin signaling pathways [8,9].

By modulating adipokine secretion from adipose tissue, PEA may enhance insulin signaling and glucose uptake in peripheral tissues like muscle and adipose tissue. Indeed, PEA may polarize adipose tissue macrophages to the M2 lean phenotype, associated with reduced inflammatory cytokines/adipokines [48]. Animal studies have suggested that PEA administration improves glucose tolerance, although not always significantly, in models of obesity and ovariectomy [48,57]. This is particularly relevant for postmenopausal women, who often experience increased insulin resistance due to hormonal changes associated with menopause [58]. Improving insulin sensitivity can reduce the risk of developing T2DM, a common consequence of metabolic syndrome.

### 4.4. Weight Management and Appetite Regulation

Weight gain and difficulty in weight loss are common concerns during menopause [59]. PEA supplementation in ovariectomized rats and obese mice resulted in a significant reduction in fat mass and adipocyte fat content compared to placebo. This aligns with the preclinical findings of PEA promoting adipocyte browning and enhancing energy expenditure [48,57].

PEA may also influence appetite regulation through its interaction with the endocannabinoid system [60]. Leptin is a hormone primarily produced by adipose tissue and plays a critical role in regulating energy balance by signaling satiety to the hypothalamus in the brain. It helps suppress appetite and promotes energy expenditure, thus contributing to weight regulation and metabolic homeostasis [61].

In the context of the mechanisms underlying the effects of PEA, an improvement in hypothalamic leptin signaling is a significant factor. PEA may enhance this signaling by increasing the phosphorylation of the signal transducer and activator of transcription 3 (STAT3) and the hypothalamic expression of leptin receptor (Or-Rb). When leptin binds to its receptors in the hypothalamus, it activates STAT3 through phosphorylation. Activated STAT3 then translocates to the nucleus, where it influences the transcription of genes involved in appetite suppression and energy expenditure. By boosting STAT3 phosphorylation and reducing the expression of two important mediators of leptin resistance—suppressor of cytokine signaling 3 (SOCS3) and protein tyrosine phosphatase 1B (PTP1B)—PEA may help overcome leptin resistance, a condition commonly associated with obesity and metabolic syndrome where the body’s responsiveness to leptin is diminished. Improved leptin signaling can lead to reduced food intake and increased energy expenditure and ultimately contribute to weight loss and better metabolic control. By improving leptin sensitivity and correcting signaling pathways involved in appetite regulation and energy homeostasis, PEA may help reduce obesity-related complications and improve metabolic health. Additionally, PEA reduces the phosphorylation of AMP-activated protein kinase-alpha (AMPK-α) in the hypothalamus. AMPK-α acts as an energy sensor; its activation in the hypothalamus is linked to increased appetite and decreased energy expenditure. By reducing AMPK-α phosphorylation, PEA further supports appetite suppression and promotes a negative energy balance. PEA also modulates the transcription of anorexigenic (leptin-responsive pro-opiomelanocortine, POMC) and orexigenic (Agouti-related protein, AgRP) factors in the hypothalamus. This modulation leads to a decreased expression of neuropeptides that stimulate appetite and an increased expression of those that suppress it, aiding in the regulation of food intake [48]. It is intriguing to hypothesize that through the positive modulation of the leptin pathway, PEA may indirectly decrease endocannabinoid synthesis and CB1 receptor expression in the hypothalamus, negatively influencing the production of the orexigenic peptide ghrelin [62]. These effects could assist postmenopausal women in managing caloric intake and achieving weight loss goals.

### 4.5. Neuroprotective Effects and Mood Regulation

Metabolic syndrome has been linked to cognitive decline and mood disorders, such as depression and anxiety [63,64]. Neuroinflammation is a contributing factor to these neurological complications [65,66]. PEA exhibits neuroprotective properties by reducing neuroinflammation and oxidative stress in the central nervous system [67]. Indeed, PEA treatment in a model of obese mice induced by a high-fat diet resulted in improved anxiety-like behaviors and a reduction in systemic inflammation by lowering serum levels of pro-inflammatory mediators such as TNF-α, interleukin-1 β (IL-1β), Monocyte Chemoattractant Protein-1 (MCP-1), and lipopolysaccharide (LPS). PEA may modulate microglial activation, leading to the decreased production of neurotoxic substances and preservation of neuronal integrity. PEA has been shown the capacity to attenuate the immunoreactivity of microglial marker Iba-1 and astrocytic marker Glial Fibrillary Acidic Protein (GFAP), leading to reduced pro-inflammatory pathways and cytokine production in both the hypothalamus and hippocampus. This effect, along with the decreased transcription of mast cell markers chymase 1 and tryptase β2 in the hippocampus, indicates a weakening of immune cell activation. Obesity-driven neuroinflammation was associated with disruption of the blood–brain barrier (BBB) in the hippocampus. PEA mitigated this by limiting albumin extravasation and restoring tight junction transcription altered by a high-fat diet [68].

For postmenopausal women who may experience mood disturbances due to hormonal changes, the neuroprotective effects of PEA could be particularly beneficial [69]. By potentially influencing neurotransmitter systems involved in mood regulation, such as serotonin and dopamine pathways, PEA may help improve mood and cognitive function. Indeed, in the amygdala, PEA increases dopamine turnover and elevated GABA levels, both of which are crucial for mood regulation. It also counteracted the overactivation of the hypothalamic–pituitary–adrenal (HPA) axis by reducing the expression of the hypothalamic corticotropin-releasing hormone and its type 1 receptor. This could alleviate some of the neuropsychological symptoms associated with both menopause and metabolic syndrome [68].

## 5. PEA in Postmenopausal Metabolic Syndrome: Potential Benefits and Limitations

### 5.1. Benefits

Integrating PEA into the management of metabolic syndrome in postmenopausal women may represent a valid support to existing interventions, especially for patients who experience significant adverse effects from standard treatments. PEA may help mitigate symptoms while reducing the reliance on medications that have undesirable side effects. Indeed, compared to pharmacological agents such as HRT or lipid-lowering drugs, PEA offers several advantages. Its endogenous nature, wide range of biological properties, and minimal side effects make it suitable for the complex symptomatology related to postmenopausal metabolic syndrome and long-term use without the significant risks associated with HRT or statins [70,71].

Non-pharmacological approaches may represent a valid option against metabolic syndrome, also emphasizing lifestyle modifications. Indeed, PEA may enhance the effectiveness of lifestyle interventions such as diet and exercise. By improving metabolic flexibility and energy utilization, PEA could amplify the benefits of physical activity and dietary modifications. This synergistic effect may lead to more significant improvements in metabolic parameters and overall health outcomes [72].

Clinical trials have indicated that PEA supplementation can reduce pain intensity and improve the quality of life in patients with various neuropathic pain [73,74]. By reducing nerve inflammation and oxidative damage, PEA may protect nerve fibers and improve nerve function [75,76]. This is particularly relevant for individuals with metabolic syndrome, as managing neuropathic pain can enhance physical activity frequency and/or intensity, which is crucial for metabolic health. Increased physical activity can improve insulin sensitivity and aid in weight management, addressing two key components of metabolic syndrome. Similarly, PEA may enhance muscle function and recovery after exercise by reducing inflammation and oxidative stress, encouraging more consistent physical activity among postmenopausal women [77,78].

PEA has an excellent safety profile, and to date, there is an absence of any documented adverse interactions with other biological molecules. An important consideration in this context is the potential for PEA to exert synergistic beneficial effects when combined with other phytochemical compounds. Synergistic interactions between PEA and certain molecules, such as resveratrol and its derivatives or β-caryophyllene, are known, particularly in the management of neuropathic pain [79,80]. In this context, exploring the synergistic or additive potential of PEA when combined with molecules already known for their beneficial effects during the peri- and postmenopausal periods is a field of interest. These benefits include mitigating the decline in estrogen levels and the consequences for bone metabolism, as well as reducing vasomotor symptoms. Examples of such molecules include isoflavones derived from soy, black cohosh, red clover, and saffron. Saffron, rich in phytochemicals—primarily crocin, a compound with strong potential for activating SIRT1—has demonstrated numerous other beneficial effects, including reducing anxiety and depression and improving sleep quality [81,82,83,84,85,86,87,88].

### 5.2. Limitations

The optimal dosing of PEA for metabolic benefits has yet to be established. Although clinical studies have used doses ranging from 300 mg to 1200 mg per day, it is still unclear what the optimal use and dosage are [89]. Its bioavailability is a consideration, as it has poor water solubility. Despite its potential, the clinical utility of PEA is limited by poor solubility and low oral bioavailability. Enhancing the solubility and absorption of PEA is crucial to maximize its pharmacological effects. One effective strategy to improve the solubility of PEA is particle size reduction through micronization and ultramicronization techniques. Micronization reduces particle size to the micrometer scale, increasing the surface-area-to-volume ratio, which enhances dissolution rates and absorption in the gastrointestinal tract. Ultramicronization further decreases particle size to the sub-micrometer range (less than one micron), significantly boosting solubility and bioavailability compared to non-micronized forms. Studies have demonstrated that ultra-micronized PEA exhibits superior pharmacokinetic profiles, leading to enhanced therapeutic efficacy [12,90].

Another approach involves formulating PEA with other bioactive compounds to create synergistic effects that may promote absorption and bioavailability. For example, combining PEA with *Equisetum arvense* L. extract (Equipea^®^) may offer additional benefits. In an in vitro model, this formulation not only significantly improved PEA absorption and distribution compared to ultra-micronized PEA but also provided complementary therapeutic effects [91]. Lipid-based delivery systems also present a viable method to enhance the bioavailability of PEA. Encapsulating PEA within liposomes or solid lipid nanoparticles can improve its solubility and protect it from degradation in the gastrointestinal environment. These carriers facilitate better lymphatic uptake and cellular absorption, leading to increased systemic availability [92,93]. 

While PEA is generally safe, monitoring is advisable, especially in individuals with pre-existing medical conditions or those taking other medications. The regular assessment of metabolic parameters—blood glucose, lipid profiles, blood pressure—is essential to evaluate the effectiveness of PEA supplementation. PEA does not appear to have significant drug interactions; however, caution is warranted when combining it with other agents that influence lipid metabolism or inflammation [94].

## 6. Conclusions

Menopause-associated hormonal changes significantly contribute to the development of metabolic syndrome in postmenopausal women, leading to increased risks of cardiovascular diseases and type 2 diabetes mellitus. PEA, through its activation of PPAR-α and modulation of inflammatory pathways, shows promise in improving metabolic parameters, reducing inflammation, enhancing insulin sensitivity, and addressing neuroinflammation, and mood disturbances. While preliminary evidence from preclinical and clinical studies is encouraging, comprehensive, large-scale clinical trials are essential to establish the efficacy, optimal dosing, and long-term safety of PEA supplementation in postmenopausal women. If validated, PEA may become an integral part of non-pharmacological strategies for managing metabolic syndrome and improving overall health outcomes in this population, potentially enhancing the quality of life by addressing both metabolic and neuropsychological aspects of menopause.
